# The antibacterial effect of sonication and its potential medical application

**DOI:** 10.1051/sicotj/2019017

**Published:** 2019-06-17

**Authors:** Srinath Kamineni, Chifu Huang

**Affiliations:** 1 Department of Orthopaedics and Sports Medicine, Elbow Shoulder Research Center, University of Kentucky 740 South Limestone Lexington 40536 KY USA; 2 Center for Oral Health Research, College of Dentistry, University of Kentucky Lexington 40503 KY USA

**Keywords:** Ultrasonic, Debridement, Diabetic, Foot, Ulcer, Tenex

## Abstract

*Introduction*: Recent applications of ultrasonic probes include cataract removal and tennis elbow treatment. Early data support the use of ultrasonic probe debridement in the treatment of recalcitrant diabetic foot ulcers. No data are available concerning the potential antibacterial properties of the clinical grade, lower energy ultrasound probes. We investigated the effect of a clinically available ultrasonic debridement probe with respect to bacterial viability.

*Methods*: A commercially available Tenex sonication machine with a Tx1 probe was used for this study. Three bacterial strains, aerobic and anaerobic, were investigated, G-negative (*Porphyromonas gingivalis*) and G-positive bacteria (*Staphylococcus aureus* and *Streptococcus gordonii*). These bacteria were cultured and tested with sonication for varying lengths of time (10, 30, 60, and 120 s). The tested bacterial samples were plated, the number of colonies on each plate counted, and the anti-bacterial effect was calculated. Statistical analysis was conducted using a one-way analysis of variance.

*Results*: Sonication exhibited a significant time-dependent antibacterial effect. Statistically significant anti-bacterial effect was observed in all three species tested. When comparing the kill rate between the control and 120 s of sonication; *S. gordonii* had a 34% kill rate, *S. aureus* had a 60% kill rate, and *P. gingivalis* had a 64% kill rate. When comparing control to all of the time intervals tested, *S. aureus* kill rate was statistically significant at all times, *S. gordonii* was statistically significant at all times above 10 s, and *P. gingivalis* was only statistically significant at 120 s.

*Conclusion*: This study demonstrates that a clinically available ultrasonic probe has an antibacterial effect against a wide spectrum of gram-positive, gram-negative, aerobic and anaerobic bacterial species. This may partially explain the dramatic healing of long-standing recalcitrant diabetic ulcers debrided with this device and may have a place in treating pathologies with bacterial mechanisms.

## Introduction

Sonication is the act of applying sound energy to agitate particles in a sample, for various purposes, such as in food processing and medical treatment. It is usually applied using an ultrasonic bath or an ultrasonic probe, colloquially known as a sonicator. Ultrasonic frequencies (>20 kHz) are usually used, leading to the process also being known as ultrasonication, and has many uses in surgery, ranging from diagnostic to therapeutic. Ultrasonication is commonly used in some types of surgery, for example in ophthalmology, cataract removal is performed by ultrasonic debridement, known as phacoemulsification, and orthopedics, where a diagnostic modality can visualize tissues, and a therapeutic modality can remove bone cement [1]. Ultrasonic technology has also been used to treat fractures and remove small cartilage tissues for orthopedic surgery. More recent applications of ultrasonic probes include focal tumor ablation [2, 3]. Most recently ultrasonic debridement of diabetic foot ulcers has successfully treated recalcitrant lesions, although the actual mechanism is unknown [4]. Diabetic ulcers are known to be etiologically influenced by mechanical, vascular, and bacterial factors. One possible hypothesis for the clinical success of ultrasonic debridement of diabetic ulcers is that there is an antibacterial effect that may decrease the poly-microbial burden that is known to exist in recalcitrant diabetic ulcers.

Sonication technology has been proven to remove bacteria from implants in-vitro with High Intensity Focused Ultrasound (HIFU). These higher frequency, higher energy waveforms, have also been proven to disrupt bacterial cell walls, but no data are currently available concerning the potential antibacterial properties of clinical grade, lower energy, ultrasound probes. Therefore, this study is designed to evaluate a clinical grade probe with a proprietary lower frequency ultra-sonic waveform, and its possible effect on bacterial integrity and growth. This study investigates a mechanism for treating diabetic ulcers; the antibacterial effect of the clinically available Tenex Tx1™ ultrasonic probe.

## Materials and methods

### Bacterial cultivation

Oral microbial species *Porphyromonas gingivalis* (ATCC 33277), *Staphylococcus aureus* (ATCC 23235), and *Streptococcus gordonii* (ATCC 10558) were purchased from the American Type Culture Collection (Manassas, VA) [5, 6]. TSBYE media and Anaerobe Broth were purchased from Oxoid Ltd. (Cambridge, UK). These three strains of bacteria were cultured under the growth conditions of 37 °C in Plas-Labs anaerobic chamber with 85% N_2_, 10% H_2_, and 5% CO_2_ (Lansing, MI).

### Antibacterial assay

Five ml of the overnight culture was placed in a test tube and tested, in triplicate. Sonication testing, with a TX1 ultrasonic probe (Tenex™, Lake Forest, CA), was performed with the active end of the probe submerged into the bacterial broth, but not touching the bottom of the test-tube. The protocol included the fluid irrigation, which is a normal part of the clinical function, was turned off, in order to prevent dilution of the bacterial broth. A thermometer was also placed in the bacterial broth test-tube, during testing to ensure the fluid temperature did not increase beyond 37.5°. Each test-tube of broth was sonicated for various time intervals (10, 30, 60, and 120 s), in triplicate. After the treatment, 3 μL of the culture solution was diluted 10^5^ times and plated to blood agar plates (Remel^®^). The plates were incubated anaerobically or aerobically, dependent on the maximal conditions for that particular species, at 37 °C for 48 h. The plates were then incubated for 48 h at 37 °C in an aerobic chamber. Then, the colony forming units (CFU) were counted for each plate. A similar procedure was performed for analysis of *P. gingivalis*, *S. aureus,* and *S. gordonii*. Species [5, 6].

### Statistical analysis

Data analysis was performed using a one-way ANOVA with multiple comparisons. Turkey’s post hoc adjustment was utilized to compare multiple groups, using SPSS v15 (IBM; Armonk, New York, USA). A *p*-value of less than 0.05 was set for statistical significance. Results are shown as mean (*M*) ± standard error (*SE*).

## Results

Sonication exhibited a significant antibacterial effect, in a time-dependent manner (Table 1). None of the species tested were completed denatured at any of the times chosen for sonication exposure. The greatest anti-bacterial effect (the greatest reduction of colony forming units) was observed at 120, 90, and 60 s, while less effect was observed at 5, 10, and 15 s. Sonication was effective in reducing the CFU counts in both G-negative and G-positive bacteria (Table 2).

**Table 1:** Time effect of sonication on the growth of planktonic bacteria analyzed by microplating. Shown are the colony counts.

	*S. gordonii*	*S. aureus*	*P. gingivalis*
Control	1720 ± 40	866 ± 18	840 ± 26
10″	1505 ± 135	825 ± 31	680 ± 11
30″	1480 ± 100	544 ± 32	572 ± 13
60″	1460 ± 20	505 ± 14	400 ± 11
120″	1150 ± 50	354.5 ± 8.5	304 ± 8

**Table 2:** Statistical analysis of the effect of Tenex sonication on various bacteria and various times.

	*S. gordonii*	*S. aureus*	*P. gingivalis*
0–120	<0.0001	<0.0001	0.0213
0–60	<0.0005	<0.0001	0.0288
0–30	<0.0009	<0.0003	0.3446
0–10	0.7152	<0.0037	0.4277

**Figure 1:**
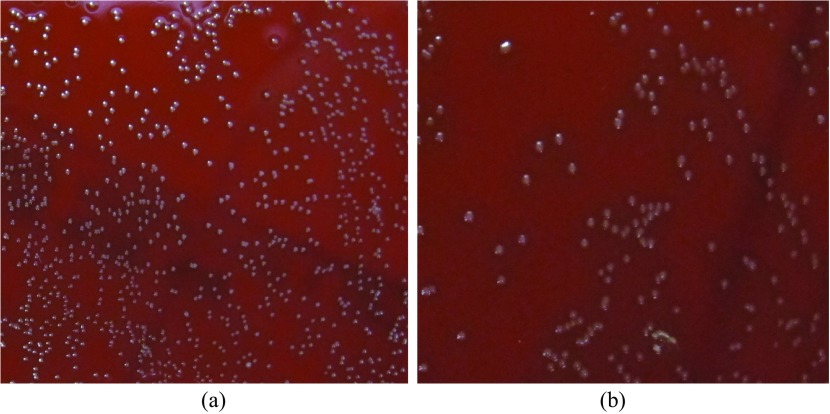
*Streptococcus gordonii*; Culture plates (a) Control. (b) 120 s.

**Figure 2:**
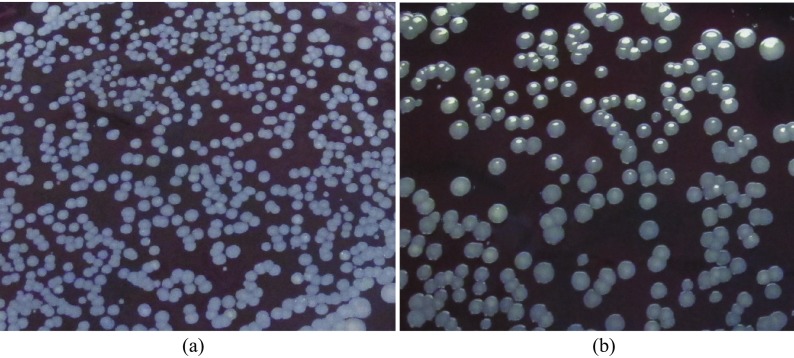
*Staphylococcus aureus*; Culture plates (a) Control. (b) 120 s.

**Figure 3:**
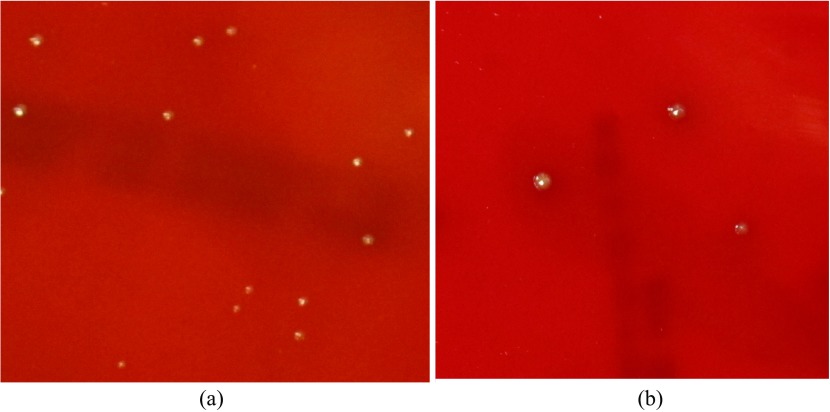
*Porphyromonas gingivalis*; Culture plates (a) Control. (b) 120 s.

Statistically significant anti-bacterial effect was observed in all species tested. When comparing the kill rate between the control and 120 s of sonication; *S. gordonii* had a 34% kill rate (Figure 1), *S. aureus* had a 60% kill rate (Figure 2), and *P. gingivalis* had a 64% kill rate (Figure 3). When comparing control to all of the time intervals tested; *S. aureus* was statistically significant at all times, *S. gordonii* was significant at all times above 10 s, and *P. gingivalis* was only significant at 120 s.

## Discussion

Ultrasonication has been studied for killing bacteria in various forms, types of bacterial species, and with various methodologies in the literature. Many studies have shown that ultrasonic energy can disrupt cell walls and diminish bacterial growth. Bacterial inactivation using ultrasound treatment was first reported in 1920s and the investigation of the mechanism of microbial inactivation began in 1960s [1, 2]. There are numerous theories available regarding the mechanism of the biocidal effects of ultrasound. Researchers believe that it is due to acoustic cavitation which causes mechanical effects and sonochemical reactions such as the generation of highly reactive radicals and molecular products such as H_2_O_2_. Although the inactivation of bacteria by high-power ultrasound is well known and extensively studied, the relationship between the effectiveness of lower ultrasound energies to inactivate bacteria and their physico-chemical properties is not yet well understood. For instance, some reports showed that gram-negative bacteria were more sensitive to ultrasonic inactivation than gram-positive bacteria, while other researchers reported no significant relationship between the gram-status of bacteria and ultrasonic inactivation [1, 2]. Our experimental data showed no significant relationship between the gram-status of bacteria and ultrasonic disruption, as the both gram-positive and gram-negative bacteria *P. gingivalis* and *S. aureus* were sensitive to Tenex ultrasonication.

This study demonstrates that a clinically available Tenex ultrasonic probe has an antibacterial effect against a wide spectrum of gram-positive, gram-negative, aerobic, and anaerobic species. Complete kill rates against any of the species was not achieved, and there is a variation in the effect based on species and the time of active sonication, with greater sonication times leading to greater kill rates. These data may help to explain the ability for ultrasonic debridement to result in dramatic healing responses of long-standing recalcitrant diabetic ulcers due to the physical diminution of the microbial load within the lesion [2, 4]. The study demonstrated that sonication technology could be used to kill bacteria; therefore a potential anti-bacterial medical application.

Limitations of the study included a small selection of bacterial species, and a small number of trials. Further studies are needed to understand this technology with respect to a wider spectrum of clinically relevant bacterial species.

In conclusion, the clinically available Tenex Tx1 probe appears to be effective at decreasing the number of bacterial colony forming units in a time- and species-dependent manner. While it was unable to eliminate all bacterial CFUs, it was effective at decreasing them by 34–64%, with sonication between 60 and 120 s. Broader spectrum studies are needed in the future, but these current results are promising for the efficacy of procedures with poly-microbial burdens, notably diabetic foot ulcers, when debrided with the Tx1 ultrasonic probe.
